# Differences in Awareness of Chinese Dietary Guidelines Among Urban and Rural Residents: A Cross-Sectional Survey in Southwest China

**DOI:** 10.3389/ijph.2023.1605344

**Published:** 2023-01-13

**Authors:** Ke Jiang, Yaqi Wen, Shengping Li, Tiankun Wang, Zhourong Li, Manoj Sharma, Zumin Shi, Yong Zhao

**Affiliations:** ^1^ School of Public Health, Chongqing Medical University, Chongqing, China; ^2^ Research Center for Medicine and Social Development, Chongqing Medical University, Chongqing, China; ^3^ Research Center for Public Health Security, Chongqing Medical University, Chongqing, China; ^4^ Department of Social and Behavioral Health, School of Public Health, University of Nevada, Las Vegas (UNLV), Las Vegas, NV, United States; ^5^ Department of Internal Medicine, Kirk Kerkorian School of Medicine, University of Nevada, Las Vegas (UNLV), Las Vegas, NV, United States; ^6^ Human Nutrition Department, College of Health Sciences, QU Health, Qatar University, Doha, Qatar; ^7^ Chongqing Key Laboratory of Child Nutrition and Health, Children’s Hospital of Chongqing Medical University, Chongqing, China

**Keywords:** rural, urban, Chinese dietary guidelines, dietary knowledge, Southwest China, nutrition survey

## Abstract

**Objectives:** This study aimed to compare the awareness of Chinese dietary guidelines (CDGs, 2016) between rural and urban areas in Southwest China and identify the factors that affect CDGs (2016) awareness.

**Methods:** This cross-sectional survey included 8,320 individuals aged 18–75 years from Chongqing, Sichuan, Guizhou, and Yunnan, China. Convenience sampling method was adopted to select the survey subjects and face-to-face surveys in each region were conducted to collect data. Descriptive statistics and generalized linear model were used to evaluate differences in awareness of CDGs among urban and rural residents and its influencing factors.

**Results:** Overall CDGs awareness was low in urban and rural areas, although the awareness rate was higher in the former than in the latter (29.1% vs. 19.9%, respectively). Region, education level, Body Mass Index (BMI), gender, income, and age are the influencing factors of CDGs awareness. Urban participants were likely to acquire relevant knowledge from social media, books/magazines, family/friends, and experts. However, rural participants were likely to acquire relevant knowledge from food sales staff (*p* < 0.05).

**Conclusion:** Rural residents are less aware of CDGs than their urban counterparts in Southwest China. Future dietary education should adopt different strategies for different populations, with considerable focus on rural residents.

## Introduction

In China, national food-based dietary guidelines (FBGDs) attempt to turn the vast and constantly incomplete evidence based on the relationships among foods, eating patterns, and health into specific, culturally appropriate, and actionable advice [1]. These dietary guidelines are reliable because they were developed based on evidence from many systematic reviews of prospective cohorts and interventional studies on the effects of nutrients, foods, and dietary patterns on risks of major chronic diseases, and involved a step-by-step process of writing, review, and revision [2, 3]. High adherence to some dietary guidelines has been associated with reduced morbidity and mortality from chronic diseases, mainly cardiovascular diseases and cancer [4, 5]. A comparative study of dietary guidelines in China, Japan, and the US has shown evidence from all respective cohort studies involved in the development of these guidelines that the risks of many chronic diseases and mortality are reduced if these guidelines are followed [6]. Studies have also found that better adherence to dietary guidelines was associated with better mental health [7–9]. For special populations in China, compliance with dietary guidelines can reduce the risk of gestational hypertension and gestational diabetes mellitus in pregnant women [10], the risk of metabolic syndrome in children aged 6–14 years [11], and the risk of cardiovascular disease among individuals with type 2 diabetes [12].

Chinese dietary guidelines (CDGs) are published by the Chinese Nutrition Society. CDGs are based on the principles of nutrition science and nutritional needs of the human body and are designed to provide suggestions on food choices and physical activities to promote the health of Chinese residents in combination with Chinese food production and supply situation and people’s living practices [13]. Given that the dietary needs and problems of the Chinese population’s dietary structure are constantly changing, CDGs have undergone four editions (i.e., 1989, 1997, 2007, and 2016), and the fifth edition was officially released in April 2022 [14, 15]. Over the past 30 years, CDGs have continued to provide residents with scientific information on healthy diets, and have played an important role in the implementation of balanced diets and nutrition in China; meanwhile, the government and relevant departments have also conducted extensive research on food and nutrition education [16]. However, the Report on Nutrition and Chronic Diseases in China (2020) and Scientific Research Report on Chinese Dietary Guidelines (2021) show that unhealthy lifestyles remain prevalent, nutrition literacy of residents should be improved, the problems of overweight and obesity are increasingly becoming prominent, and illnesses and incidences of chronic diseases are increasing [17, 18]. These findings show that the practice of dietary guidelines among Chinese residents is unsatisfactory.

A few studies have been conducted on awareness of the different CDGs editions in different regions. A national cohort study from 2004 to 2011 showed that adults’ awareness rates of the dietary guidelines were 7.8%, 11.9%, 14.6%, and 24.4%, respectively, in nine provinces in 2004, 2006, 2009, and 2011, showing a rising trend [19]. A cross-sectional study in Guangxi province found the awareness rate of dietary guidelines was only 8.3% for urban and rural residents in 2015. And there were significant differences in genders, age groups, occupations, and educational groups [20]. Both studies evaluated residents’ awareness of CDGs (2007). The reasons for the difference in results were the different survey regions and the different definitions for awareness of CDGs in the two studies. In the former study, awareness was judged by asking 12 questions about specific items in the CDG, while in the latter study, awareness was judged by only one question: “Do you know CDG?” There was also a study investigated the awareness of CDGs (2016) among residents in Zhengzhou city, which found that the awareness rate of CDGs (2016) was 40.5%, although this study was also judged by the single question of whether they knew about CDGs [21].

In China, urban areas have higher socioeconomic levels than rural areas, and previous studies have documented trends and gaps in health disparities of Chinese rural *versus* urban adults [22, 23]. Likewise, there was a huge difference in nutrition and health-related knowledge, attitudes, and behaviors between urban and rural residents [24, 25]. Hence, it is reasonable to assume that there may also be gaps in CDGs awareness and practices between rural and urban areas, which is what our study aims to demonstrate.

Considering that 1) different studies had different definitions for awareness of CDGs 2) a few studies have explored differences in awareness of CDGs (2016) between rural and urban areas. 3) no studies have described the awareness and influencing factors of dietary guidelines in Southwest China. Thus, the purposes of this study were as follows: 1) to compare the awareness of CDGs (2016) between rural and urban areas in Southwest China and 2) to identify the factors that affect CDGs (2016) awareness. We used a series of questions to determine whether participants truly were aware and understood the CDGs, rather than through participants’ self-reports. This study could provide a reference for the promotion and implementation of the latest edition of CDGs in consideration of residence and other demographic characteristics.

## Methods

### Study Design and Sample

A cross-sectional survey was conducted between February and May 2021. Convenience sampling method was adopted to select the survey objects. We recruited 252 screened and trained university students from eight universities as investigators in Chongqing, Sichuan, Guizhou, and Yunnan. Investigators used paper questionnaires to conduct face-to-face surveys in families and communities in each region. Inclusion criteria of the participants were as follows: 1) local residence for at least 1 year, 2) aged 18–75 years, and 3) informed consent and cooperation in completing the questionnaire. Those who were unable to cooperate with the completion of the survey owing to illness or other factors were excluded. The sample size required for the study was estimated by the sample size calculation formula of the cross-sectional study. $n={\left(\frac{Z_{\alpha /2}}{d}\right)}^{2}*p\left(1-p\right)$ . A previous study showed that in 2015, the knowledge rate of dietary nutrition among Chinese adults was 21.1% [26]. So, we set *p* = .211, *q* = 1-p = .789, and margin of error d = .10 × p = .021, Z_α/2_ = 1.96. The calculated sample size was 1450 for each region and 5800 in total. Considering sampling error and invalid questionnaires, an additional 20% was added to the estimated sample size, and the final sample size required was calculated to be 6960. In our study, a total of 8,535 residents participated. After excluding outliers and missing values, 8,320 participants (61.2% urban residents) were included in the analysis. All participants signed informed consent before the investigation, and the study was approved by the Ethics Committee of Chongqing Medical University on 13 July 2020 (approval number: 2021041).

### Data Collection

The survey was a national food culture survey conducted by the Chinese Nutrition Society, and the questionnaire was designed by the Chinese Nutrition Society Science Communication and Popularization Experts. Based on the data collected from the pilot study, the Cronbach’s α coefficient was calculated to be 0.825, which was greater than 0.80, indicating that the questionnaire had acceptable reliability [26]. The questionnaire consists of two parts: sociodemographic characteristics and basic knowledge of CDGs. Sociodemographic characteristics included 1) gender (male/female), 2) age, 3) height (self-reported), 4) weight (self-reported), 5) ethnicity (Han/minority), 6) residence (rural/urban), 7) region (Guizhou Province/Yunnan Province/Sichuan Province/Chongqing City), 8) occupation (laborers/students/intellectuals), 9) education (elementary and below/junior high school/senior high school/junior college/undergraduate/master’s or above), (10) average monthly household income (3000 RMB and below/3000–4999 RMB/5000–9999 RMB/10,000–19,999 RMB/20,000–39,999 RMB/40,000 RMB and above), and (11) channels for obtaining relevant knowledge of CDGs (food sales staff/experts/families or friends/books or magazines/radio or TV/social media).

Basic knowledge of CDGs included the following aspects: 1) daily cooking oil intake recommended by the guidelines, 2) daily salt intake recommended by the guidelines, 3) daily milk and dairy products intake recommended by the guidelines, 4) daily water intake recommended by the guidelines, 5) daily vegetables intake recommended by the guidelines, 6) number of daily food types recommended by the guidelines, 7) the best way to obtain calcium, 8) reasonable combination for a nutritious breakfast, 9) nutrients associated with hypertension, 10) safe pickling time for pickles, 11) foods that prevent cardiovascular diseases, and 12) best food sources of iron.

### Data Process

According to previous relevant studies [24, 27, 28] and baseline variables that showed a significant relationship with the outcome in the univariate analysis (Supplementary Table S1), the following eight demographic characteristics were identified as explanatory variables: region, occupation, education, BMI, gender, income, ethnicity and age.

Age was divided into three groups: youth (18–44 years), middle aged (45–59 years), and elderly (60–75 years) [29]. Body mass index (BMI) was calculated by self-reported height and weight (weight/height^2^), and divided into underweight (<18.5 kg/m^2^), normal (18.5 kg/m^2^ ≤ BMI <24 kg/m^2^), overweight (24 kg/m^2^ ≤ BMI <28 kg/m^2^), and obese (BMI ≥28 kg/m^2^) [30]. Education was classified into low (junior high school and below), medium (senior high school/junior college), and high (college/bachelor’s degree and above). Average monthly family income was divided into four groups: below 5,000 RMB, 5,000–9,999 RMB, 10,000–19,999 RMB, and 20,000 RMB and above. Occupation involves over 10 industries, and we divided occupations into three groups to prevent having markedly few persons in each category: laborers (including mill workers, farmers, herdsmen, fishermen, salespeople, contractors), intellectuals (doctors, nurses, teachers, officials, public servants, lawyers, managers, office clerks, reporters), and students.

For basic knowledge of CDGs, one point for each correct answer and no point for wrong answers were given. Total scores for the preceding questions ranged from 0 to 12. A score above 6 (50%) was considered good awareness of CDGs, while six and below were poor. Higher scores indicated better awareness.

### Statistical Analysis

Frequency and proportions (%) were used to describe categorical variables and mean ± standard deviation (SD) was utilized to describe continuous variables. Chi-square tests were conducted to show the differences in awareness of CDGs between rural and urban regions, as well as other social demographic characteristics. An analysis of variance (ANOVA) test was used to examine age difference between urban and rural areas. A generalized linear model (GLM) was used to assess the association between demographic characteristics and awareness level of CDGs. Graph prism was utilized to visualize the channels for obtaining relevant knowledge of CDGs. All data were entered into EpiData3.1 and STATA version 17.0 (STATA Corporation, College Station, TX, United States) was used in all analyses. Statistical significance was considered when *p* < .05 (two-sided).

## Results

### Participants’ Characteristics

Of the 8,535 participants who answered the questionnaires, 8,320 were considered eligible and the questionnaire validity rate was 97.48%. Sociodemographic characteristics of the participants are presented in Table 1. Average age was 35.1 ± 13.9 years, and 46.1% of the participants were females. The highest number of participants came from Chongqing (34.5%), followed by Guizhou (27.8%), Sichuan (20.1%), and Yunnan (17.6%). Over half of the participants were laborers (56.6%), with a high percentage of rural participants being laborers (64.3%) as well. Difference between urban and rural areas was also reflected in education, with only 36.8% of rural participants having high education. Most rural participants had an average monthly household income of below 5000 RMB (53.4%), higher than urban participants (32.9%). Overall CDGs awareness was low in urban and rural areas, although the awareness rate was higher in urban than in rural areas (29.1% vs. 19.9%, respectively).

**TABLE 1:** Distribution by Residence across demographic characteristics (China, 2021).

Factor	Total	Rural	Urban	*p*-value
N	*n* = 8,320	*n* = 3231	*n* = 5089	
Age (years)	35.1 (13.9)	35.3 (14.3)	35.0 (13.6)	0.240
Region				
Guizhou	2316 (27.8%)	969 (30.0%)	1347 (26.5%)	<0.001
Yunnan	1464 (17.6%)	575 (17.8%)	889 (17.5%)	
Sichuan	1669 (20.1%)	660 (20.4%)	1009 (19.8%)	
Chongqing	2871 (34.5%)	1027 (31.8%)	1844 (36.2%)	
Occupation				
Laborer	4711 (56.6%)	2076 (64.3%)	2635 (51.8%)	<0.001
Student	1913 (23.0%)	768 (23.8%)	1145 (22.5%)	
Intellectual	1696 (20.4%)	387 (12.0%)	1309 (25.7%)	
Education				
Low	2538 (30.5%)	1389 (43.0%)	1149 (22.6%)	<0.001
Medium	1711 (20.6%)	652 (20.2%)	1059 (20.8%)	
High	4071 (48.9%)	1190 (36.8%)	2881 (56.6%)	
BMI				
Underweight	942 (11.3%)	377 (11.7%)	565 (11.1%)	0.550
Normal	5247 (63.1%)	2029 (62.8%)	3218 (63.2%)	
Overweight	1755 (21.1%)	669 (20.7%)	1086 (21.3%)	
Obese	376 (4.5%)	156 (4.8%)	220 (4.3%)	
Gender				
Male	3832 (46.1%)	1609 (49.8%)	2223 (43.7%)	<0.001
Female	4488 (53.9%)	1622 (50.2%)	2866 (56.3%)	
Income				
5,000 below	3402 (40.9%)	1726 (53.4%)	1676 (32.9%)	<0.001
5,000–9,999	2623 (31.5%)	934 (28.9%)	1689 (33.2%)	
10,000–19,999	1589 (19.1%)	410 (12.7%)	1179 (23.2%)	
20,000 above	706 (8.5%)	161 (5.0%)	545 (10.7%)	
Ethnicity				
Han	7371 (88.6%)	2849 (88.2%)	4522 (88.9%)	0.340
Minority	949 (11.4%)	382 (11.8%)	567 (11.1%)	
Age				
Youth	5311 (64.0%)	2033 (63.1%)	3278 (64.6%)	0.004
Middle aged	2532 (30.5%)	980 (30.4%)	1552 (30.6%)	
Elderly	457 (5.5%)	211 (6.5%)	246 (4.8%)	
Awareness of CDGs				
Good	6194 (74.4%)	2587 (80.1%)	3607 (70.9%)	<0.001
Poor	2126 (25.6%)	644 (19.9%)	1482 (29.1%)	

Data are presented as mean (SD) for continuous measures, and n (%) for categorical measures.

### Urban-Rural Differences in Responses to Each Question

Comparison of the correct rates for each question on CDGs between urban and rural participants is presented in Table 2. Overall, the highest (reasonable combination for a nutritious breakfast) and lowest (safe pickling time for pickles) correct answer rates were 59.8% and 12.1%, respectively. In 10 of the 12 questions, urban participants had higher correct answer rates (*p* < .05). For the question on safe pickling time for pickles, there was no statistical difference in the correct answer rate between urban and rural participants (*p* = .720). For the question on the best food source of iron, rural participants had higher correct answer rates (*p* < .05).

**TABLE 2:** Comparison of Chinese dietary guidelines knowledge between urban and rural participants (China, 2021).

Factor	Total	Rural	Urban	*p*-value
N	8,320	3231	5089	
Knowledge on daily food intake recommended by CDGs				
Cooking oil	2576 (31.0%)	945 (29.2%)	1631 (32.0%)	0.007
Salt	2773 (33.3%)	940 (29.1%)	1833 (36.0%)	<0.001
Milk and dairy products	1893 (22.8%)	637 (19.7%)	1256 (24.7%)	<0.001
Water intake	2373 (28.5%)	881 (27.3%)	1492 (29.3%)	0.043
Vegetables intake	2738 (32.9%)	990 (30.6%)	1748 (34.3%)	<0.001
Numbers of daily food types recommended by the guideline	1328 (16.0%)	483 (14.9%)	845 (16.6%)	0.044
Best way to get calcium	3958 (47.6%)	1243 (38.5%)	2715 (53.4%)	<0.001
The reasonable combination for a nutritious breakfast	4977 (59.8%)	1767 (54.7%)	3210 (63.1%)	<0.001
Nutrients associated with hypertension	3492 (42.0%)	1132 (35.0%)	2360 (46.4%)	<0.001
Safe pickling time for pickles	1005 (12.1%)	385 (11.9%)	620 (12.2%)	0.720
Foods that prevent cardiovascular diseases	3950 (47.5%)	1334 (41.3%)	2616 (51.4%)	<0.001
The best food source of iron	1501 (18.0%)	646 (20.0%)	855 (16.8%)	<0.001

Data are presented as n (%) for categorical measures.

### Identifying Factors Affecting CDGs Awareness

GLM was utilized to identify the influencing factors of CDGs awareness and residence subgroups (Table 3). Note that such factors as region, occupation, education level, BMI, gender, income, and age were the influencing factors of CDGs awareness. For the residence subgroups, students (OR: 1.29; CI: 1.13–1.47), intellectuals (OR: 1.40; CI: 1.25–1.57), those with medium (OR: 1.75 CI: 1.47–2.08) or high (OR: 2.14 CI: 1.80–2.56) level of education, obese participants (OR: 0.74; CI: 0.57–0.97), females (OR: 1.23 CI: 1.12–1.34), those with an average monthly household income between 5,000 and 9,999 RMB (OR: 1.13; CI: 1.01–1.27), middle aged (OR: 1.50; CI: 1.36–1.67), and elderly (OR: 1.58; CI: 1.25–2.00) participants in urban regions were likely to have good awareness of CDGs, compared with laborers, those with low level of education, normal BMI participants, males, those with an average monthly household income below 5000 RMB and young participants. However, only intellectuals (OR: 1.35; CI: 1.09–1.68), those with medium (OR: 1.47 CI: 1.17–1.85) or high (OR: 1.82 CI: 1.42–2.32) level of education, females (OR: 1.27 CI: 1.11–1.47) in rural regions were aware, compared with laborers, those with low level of education and males. BMI, income, and age were not associated with CDGs awareness.

**TABLE 3:** Association between sociodemographic factors and Chinese dietary guidelines awareness (OR, 95%CI) (China, 2021).

Factor	Total	Rural	Urban
Region			
Guizhou (Ref)			
Yunnan	1.47 (1.30–1.65)***	1.92 (1.55–2.37)***	1.28 (1.11–1.47)***
Sichuan	1.47 (1.31–1.65)***	1.67 (1.34–2.07)***	1.39 (1.21–1.58)***
Chongqing	1.41 (1.27–1.56)***	1.63 (1.33–1.99)***	1.31 (1.16–1.49)***
Occupation			
Laborer (Ref)			
Student	1.23 (1.10–1.38)***	1.13 (0.92–1.38)	1.29 (1.13–1.47)***
Intellectual	1.39 (1.26–1.54)***	1.35 (1.09–1.68)***	1.40 (1.25–1.57)***
Education			
Low (Ref)			
Medium	1.69 (1.47–1.94)***	1.47 (1.17–1.85)***	1.75 (1.47–2.08)***
High	2.07 (1.80–2.39)***	1.82 (1.42–2.32)***	2.14 (1.80–2.56)***
BMI			
Normal (Ref)			
Underweight	0.96 (0.86–1.08)	1.03 (0.85–1.26)	0.93 (0.81–1.07)
Overweight	0.99 (0.90–1.09)	1.00 (0.83–1.21)	0.97 (0.87–1.08)
Obese	0.75 (0.60–0.95)**	0.76 (0.50–1.16)	0.74 (0.57–0.97)**
Gender			
Male (Ref)			
Female	1.24 (1.15–1.34)***	1.27 (1.11–1.47)***	1.23 (1.12–1.34)***
Income			
5,000 below (Ref)			
5,000–9,999	1.12 (1.03–1.23)**	1.09 (0.93–1.28)	1.13 (1.01–1.27)**
10,000–19,999	1.03 (0.93–1.15)	0.90 (0.73–1.12)	1.07 (0.95–1.20)
20,000 above	1.05 (0.92–1.20)	1.01 (0.75–1.36)	1.05 (0.90–1.23)
Ethnicity			
Han (Ref)			
Minority	0.99 (0.88–1.11)	0.98 (0.79–1.22)	1.00 (0.87–1.15)
Age			
Youth (Ref)			
Middle aged	1.36 (1.24–1.49)***	1.02 (0.84–1.24)	1.50 (1.36–1.67)***
Elderly	1.26 (1.03–1.56)**	0.73 (0.48–1.13)	1.58 (1.25–2.00)***

OR, odds ratio; CI, confidence interval; ***p < 0.01, **p < 0.05, *p < 0.1.

### Channels for Obtaining Relevant Knowledge of CDGs

**FIGURE 1:**
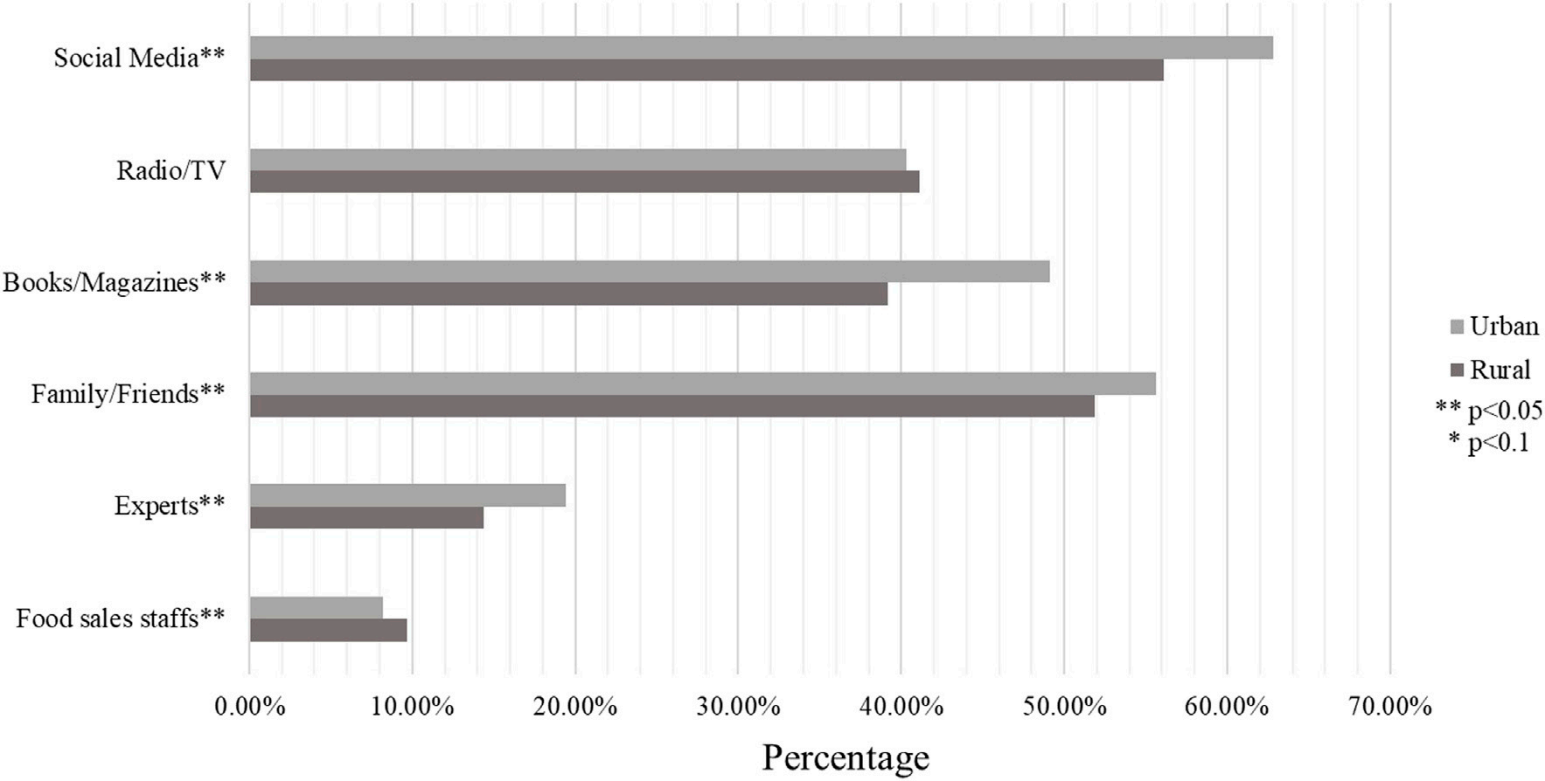
Channels for obtaining relevant knowledge of Chinese dietary guidelines (China, 2021).

Figure 1 illustrates the proportion of urban and rural participants obtaining relevant knowledge of CDGs on six channels. Overall, social media was the most common way for urban and rural participants to acquire relevant knowledge, whereas food sales staff was the least common way. Urban participants were likely to acquire relevant knowledge from social media, books/magazines, families/friends, and experts. However, rural participants were likely to acquire relevant knowledge from food sales staff (*p* < 0.05). No statistical difference exists between the proportion of rural and urban participants who chose to obtain relevant knowledge from radio/TV.

## Discussion

Six years have passed since the fourth edition of CDGs was published, but only a few studies have explored whether or not it is known and practiced by residents. The objective of this study was twofold: to compare the awareness of CDGs between rural and urban areas in Southwest China and to identify the factors affecting CDGs awareness. Our study showed that awareness of CDGs in Southwest China is extremely low, with an awareness rate of only 25.6%. It is similar to the 21.1% awareness rate reported from nine provinces in another Chinese study [26], and lower than the 59.6% awareness rate of core recommendations of CDGs reported in a survey conducted in Wuhan [24]. Moreover, rural residents’ awareness rates were significantly worse than that of urban residents. CDGs contain a range of healthy dietary recommendations, including advice on eating plenty of grains, vegetables, fruits, dairy products and soya foods, moderate amounts of fish, poultry, eggs and lean meat, and limiting fat and salt intake [31]. Hence, urban and rural differences in awareness of CDGs also reflect differences in dietary knowledge. Our findings are consistent with what has been found in previous study. A study based on the China Nutrition Survey database has found that participants with low education levels or living in rural or western areas are unaware of the China Food Pagoda or CDGs [32]. Another cross-sectional study has focused on diet-related knowledge, attitudes, and behaviors (KABs) of Chinese residents, and has found that diet-related KABs are poor among adult Chinese residents and that rural residents report significantly worse diet-related KABs than urban residents [25]. Residents of many countries have insufficient knowledge of their own dietary guidelines. A study in the Philippines has found that 92.7% of urban housewives are aware of nutritional guidelines for Filipinos (NGF), whereas only 4% of rural housewives are aware [33]. A survey conducted in United States has indicated that 58% of the participants claim to have heard of the food guide pyramid (FGP), but just 13% claim to have comprehended it [34]. These results indicate the importance of diet-related health education in China’s rural areas and not just the promotion of dietary guidelines publicity. Future studies should utilize theoretical frameworks that advocate behavior change over and above simply knowledge acquisition [35].

Our study also found that urban residents have a higher proportion of correct responses to knowledge of CDGs on almost every question compared with rural residents, except for Q10 (safe pickling time for pickles) and Q12 (best food source of iron). No statistical difference exists in the correct rate of the Q10 among rural and urban residents. Pickle is a high-salt, fermented food made by lactic acid bacteria using vegetable products as starting material. It is highly popular in China owing to its distinctive flavor and texture [36]. However, considerably short or long fermentation time of pickle will lead to high nitrite content, which may affect health outcomes [37]. Therefore, extensive health education efforts should be exerted to teach urban and rural residents the proper way to make pickles. Interestingly, rural residents were more likely to get Q12 correct than urban residents. The majority of the participants thought that the main source of iron is spinach or stir-fried vegetables in an iron pan, and only 20.0% of rural residents and 16.8% of urban residents correctly selected animal offal as the best source of iron supplement. The possible reason of this opposite difference is that various information sources are currently flooded with pseudo-science propaganda on iron supplements [38]. Compared with urban residents, rural residents have less access to health information from such sources as primary care providers, specialists, blogs, and magazines; and less use of search engines, possibly leading them to be less influenced to make the right choices by these pseudoscientific propaganda [39].

In regression analyses, awareness of CDGs differed across region. We found that participants from Guizhou Province have lower awareness of CDGs compared with those from the other three provinces. Xu et al. [40] concluded significant differences in dietary knowledge among individuals with different incomes and from different regions. It may relate to economic level of different provinces. Moreover, women have a better awareness rate of CDGs than men in rural and urban areas. The possible reason is that wives in China are mainly responsible for preparing food at home, and they will learn more dietary knowledge to promote healthy eating in the family. This situation is consistent with the results of a study published in 2019, which indicated that females have better adherence to recommended diets and better diet quality [41]. So, women should be more empowered in the domains of social support, and familial rights, which can be a significant pathway for enhancing the food security and the vulnerability of their households. As more bargaining power of women over the utilization of resources could result in a good quality of food choices [42, 43]. Participants with high-level education have a good knowledge of CDGs probably because they tend to focus considerably on learning dietary knowledge and preventing health risks [44]. By contrast, participants who have low-level education did not know of CDGs and did not proactively look for nutrition knowledge were less likely to have adequate dietary knowledge literacy [32]. This evidence shows that education level was a major factor affecting the awareness rate of respondents’ nutritional knowledge; the higher the education level, the higher the awareness rate of the related knowledge [45]. Furthermore, individuals with a lower level of education benefit more from increasing dietary knowledge. Therefore, people’s dietary knowledge for improving the health of the low-education group must be enhanced [46].

Our results have some significant differences that exist only in urban and not in rural areas. For example, awareness of CDGs in middle-aged and elderly people is generally higher than the youth in urban areas. However, focus groups and key informant interviews in New Zealand have revealed that the elderly, parents, and children and adolescents have limited awareness of FBDG [47]. Thus, appropriate food nutrition education strategies should be formulated according to the characteristics of age groups [48]. Those with obesity have better awareness of CDGs than normal, underweight, and overweight ones. Although other studies have defined opposite results, another research showed that people with high BMI (obesity) likely have consumers’ (incorrect) dietary knowledge [49]. Another study has revealed that BMI has no relationship with dietary knowledge [50–52]. Household income level is one of the important factors affecting the practice of dietary guidelines by urban residents [53]. Our study also found that those with medium income (5,000–9,999 RMB) have a higher awareness of CDGs than low- and high-income individuals. The possible reason is that people with low and high incomes spend significant time making money and disregarding healthy eating. Another study found a positive correlation between the income of adult residents and dietary quality in 15 provinces in China [54]. Meanwhile, a study has shown no statistical significance between economic situation and awareness rate of CDGs [55]. Hence, we should take corresponding measures according to the specific situation of different regions. The absence of significant differences in these variables in rural areas is likely caused by the generally lower awareness of CDGs among rural residents, irrespective of their classification. Thus, there was a need to close the urban-rural gap and create interventions that were specifically aimed at rural residents. In rural or underdeveloped areas, the government should step up its effort to promote diet-related health education that fosters behavioral change of the poor toward healthy diets [25] These actions will undoubtedly provide concrete ways to achieve sustainable food security and nourishment, especially for the underprivileged [56].

We also investigated how rural and urban residents often obtained knowledge of CDGs. Note that with the development of Internet technology and the popularity of smartphones, social media has become the main platform for people to obtain information on nutrition and health [57]. In addition, a higher proportion of urban residents obtained this information through social media than rural residents. This result is also consistent with the higher smartphone penetration rate in urban China [58]. Compared with rural residents, urban residents are more likely to obtain knowledge of CDGs through books/magazines and expert lectures. This finding reflects the huge gap in education between rural and urban residents. In China, less than half of rural junior middle school students advanced to academic high school either because they do not pass competitive admission tests or they choose not to pursue high school at all [59]. Over 70% of urban students are admitted to college compared with under 5% of rural students [60]. Moreover, rural students have worse educational resources in their schools and home reading environments [61, 62]. Therefore, rural residents have limited access to these reading materials and experts. Lastly, a high proportion of rural participants willing to acquire knowledge from food sales staff may not be bad, but there is also the risk of sales fraud. Statistical information showed people in rural areas are among the main victims of telecom network fraud [63]. Food sales staff members may also mislead consumers by false claim of functions of certain foods. Therefore, the way of nutrition knowledge popularization in rural areas should be broadened, particularly in becoming aware of being cheated.

Barriers are present to the spread of CDGs. Residents have some awareness of the major concrete concepts in FBDG but have relative difficulty grasping abstract notions and concrete values, such as portion size and quantity [64]. For example, the terms “low sugar diet” or “low-fat diet” were thought to be imprecise and difficult to quantify [65]. Meanwhile, such terms as “grams” and “milliliter” are difficult to implement because only a few residents use scales and measuring cylinders at home. A suggestion was for residents to be educated in such ways as removing chicken skin rather than eating less fat, and visual examples (solid fat vs. oil) rather than professional terms (saturated fat vs. unsaturated fat). Moreover, residents’ perspective should be emphasized rather than that of scientists, and that the former should not be asked to become nutritionists [66, 67]. Sociocultural influences on lifestyle behaviors should also be considered in CGD promotion. To be able to target particular populations, groups must be distinguished at least by residence. Hence, future nutrition guidelines are recommended to provide guidance that assists specific population groups affected by the different problems [68].

This study was the first cross-sectional research with a large-scale sample exploring the current situation of CDGs (2016) awareness in Southwest China. However, there are several limitations that should be acknowledged. First, given that our survey was conducted before CDGs (2022) were issued, we were unable to obtain information on residents’ knowledge of the latest edition of the dietary guidelines. Second, CDGs contain numerous entries and information, even though the questionnaire design was guided by experts in the field of eating behavior or nutrition in the Chinese Nutrition Society, it was not possible to cover all contents of CDGs. Third, despite the professional training of our investigators, the generalizability of the results to the population was somewhat limited by the use of convenience sampling method in this study. Lastly, the researcher’s capacity to draw direct causal inferences is diminished by the use of cross-sectional survey data.

### Conclusion and Policy Recommendations

This study is conducted to compare the awareness of CDGs (2016) between rural and urban areas in southwest China and identify the factors that affect CDGs (2016) awareness.

The overall findings show rural residents are less aware of CDGs than their urban counterparts in Southwest China. Region, education level, BMI, gender, income, and age are the influencing factors of CDGs awareness. Rural and urban residents also prefer different channels to obtain knowledge about CDGs. Urban participants were likely to acquire relevant knowledge from more modern and authoritative channels like social media, books or experts. However, rural participants were likely to acquire relevant knowledge from food sales staff which may risk exposure to sales fraud.

This study reveals a significant urban-rural gap in dietary and nutrition-related knowledge, which highlights the importance of diet-related health education in China’s rural areas and not just the promotion of dietary guidelines publicity. CDGs awareness, i.e., nutrition and health knowledge level vary across populations with various demographic characteristics, suggesting that future dietary education should consider residents’ perspective rather than that of scientists. Medical practitioners, nutritionists and educators should go deep into communities, schools and economically disadvantaged regions to adopt different strategies for different populations, with more focus on males, laborers, young people, less educated people, obese people, and people on low incomes. Given that rural residents still have relatively poor channels for obtaining relevant knowledge of CDGs, there is a need to continue to improve access to modern information communication technologies and specialists in rural areas by giving them multiple subsidy mechanisms to improve their access. The findings and recommendations of our study could provide a reference for the promotion and implementation of the latest edition of CDGs in consideration of residence and other demographic characteristics.
