# Supplementary material for: Differences in Awareness of Chinese Dietary Guidelines Among Urban and Rural Residents: A Cross-Sectional Survey in Southwest China
Source: Int J Public Health. 2023 Jan 13;68:1605344. doi: 10.3389/ijph.2023.1605344 (PMC9879960; doi:10.3389/ijph.2023.1605344)
Supplement: Supplementary file 1 [file Table1.DOCX]

***Supplementary Table S1*** ***Univariate analysis of association between sociodemographic factors and Chinese dietary guidelines awareness, China, 2021***

|  |  | **Awareness of CDGs** | |  |
| --- | --- | --- | --- | --- |
| **Factor** |  | **Poor** | **Good** | **p-value** |
| N |  | n=6194 | n=2126 |  |
| Region | Guizhou | 1904 (30.7%) | 412 (19.4%) | <0.001 |
|  | Yunnan | 1032 (16.7%) | 432 (20.3%) |  |
|  | Sichuan | 1181 (19.1%) | 488 (23.0%) |  |
|  | Chongqing | 2077 (33.5%) | 794 (37.3%) |  |
| Occupation | Worker | 3816 (61.6%) | 895 (42.1%) | <0.001 |
|  | Student | 1340 (21.6%) | 573 (27.0%) |  |
|  | Intellectual | 1038 (16.8%) | 658 (31.0%) |  |
| Education | Low | 2181 (35.2%) | 357 (16.8%) | <0.001 |
|  | Medium | 1298 (21.0%) | 413 (19.4%) |  |
|  | High | 2715 (43.8%) | 1356 (63.8%) |  |
| BMI | Thinness | 683 (11.0%) | 259 (12.2%) | <0.001 |
|  | Normal | 3858 (62.3%) | 1389 (65.3%) |  |
|  | Overweight | 1340 (21.6%) | 415 (19.5%) |  |
|  | Obese | 313 (5.1%) | 63 (3.0%) |  |
| Gender | Male | 2994 (48.3%) | 838 (39.4%) | <0.001 |
|  | Female | 3200 (51.7%) | 1288 (60.6%) |  |
| Income | 5000 below | 2696 (43.5%) | 706 (33.2%) | <0.001 |
|  | 5000-9999 | 1872 (30.2%) | 751 (35.3%) |  |
|  | 10000-19999 | 1131 (18.3%) | 458 (21.5%) |  |
|  | 20000above | 495 (8.0%) | 211 (9.9%) |  |
| Ethnicity | Han | 5482 (88.5%) | 1889 (88.9%) | 0.660 |
|  | Other | 712 (11.5%) | 237 (11.1%) |  |
| Age | Youth | 3927 (63.6%) | 1384 (65.3%) | <0.001 |
|  | Middle aged | 1877 (30.4%) | 655 (30.9%) |  |
|  | Elderly | 375 (6.1%) | 82 (3.9%) |  |

Chi-square tests were conducted to show the differences in awareness of CDGs between sociodemographic factors.

Data are presented as n (%) for categorical measures.
